# The Country Doctor

**Published:** 1853-04

**Authors:** 


### ECLECTIC DEPARTMENT,

### AND SPIRIT OF THE MEDICAL PERIODICAL PRESS.

*The Country Doctor.*—Some of our contemporaries pride themselves upon never quoting anything from "the secular press;" but we have always preferred to adopt the wiser course suggested by the motto of the Southern Medical Journal—"je prends le bien où je le trouve"—and in so doing do not feel that any one's professional dignity has been injured, or any one's professional robe sullied by contact with the common herd. Acting on these principles, we give below an article from the Knickerbocker, entitled "The Country Doctor; a faithful Autobiography. By Glauber Saultz, M. D." Perhaps all our readers can narrate facts which surpass these; we are sure almost all can equal them; but then it is easier to laugh at another's misfortunes than our own, and "Doctor Saultz's" feelings will not be hurt if every one laughs as hard as it will answer for a dignified M. D.; neither will he make a charge for this visit:

I had stumped about the country for a dozen years or so, in the same equipage, having wonderful success in curing "cases," but half the time cheated out of the credit of it by catnip tea. I took a notion to cast up my books to see how rich I was, and what could be made of outstanding accounts. It cost a great many evenings of hard work to arrive at the knowledge that, all debts being paid I was not worth a "brass farthing"—not a red cent. Notwithstanding all the lucrative cases of typhus which I had managed, I remained poor. I believe that people in the city pay their fees with alacrity, because the charges are exorbitant. When a bill for a hundred dollars, for looking two or three times at a sick child, is presented to one who lives in a well-furnished house in the upper part of the town, the very largeness of the demand is a delicate compliment upon his ability to pay. The man of the house sits down at a handsome secretary, and draws out a clean check for the full amount, saying, "Doctor, you are very moderate: now that Jacky is out of the woods, come in, in a sociable way."

As soon as the messenger is gone, the *pater-familias* exclaims, "What an outrageous bill! It is an expensive luxury to be sick." However, it has its advantages to be attended by a fashionable doctor, as it has to worship in a fashionable church. On one occasion I was called in midsummer to attend a sick man on the sea-shore. After several days, his family physician, the renowned Doctor Jallaps, arrived from the city, and the patient was soon after on his legs, no thanks to me, and ready for the surf.

"How much are you going to charge him?" said Doctor Jallaps.

"Twenty-five dollars," said I.

"Poh!" said he, "make it a hundred. He expects it."

"If he expects it," said I, "it would give me great pain to disappoint his expectations;" whereupon I acted advisedly, and received an honored check for a round C. on the Phœnix Bank.

On another occasion, when attending one of my own patients in the same vicinity, while crossing the "big bridge," when the tide was up, I came near being drowned. My sulky was soon afloat, but the horse, being a good swimmer, reached the opposite bank. Now, besides risking my own life, I fairly dragged the patient from the very gates of death. I got him out of a bilious-remittent, drove the jaundice out of his skin, and when I came to ask him for ten dollars, he blackguarded me like a chicken-stealer, and would never employ me again. The fact is, that people in the country abhor taxes and a doctor is the worst of publicans. To be sick they think is a dead loss, which they unchristianly grumble at; but to have to pay for being cured irritates them beyond measure. Oh! how meek they are when they lie prostrate in a burning fever—when their teeth chatter, and the whole house jars with their shaking ague! Oh! how welcome the latch is lifted up to admit you when life seems to hang upon a hair! But get them on their legs, and the first thing which they forget will be that they were ever on their backs. If many of them do pay you, it is under protest, procrastinating the settlement to a time when the account might be outlawed, clipping down the fair proportion of a just bill, and giving you the most ragged representative of money.

I say that when I came to overhaul my accounts, I was not worth anything, and therefore arrived at the conclusion that it was high time to marry a wife who would take care of my money. I did so, and found my condition better, but for some years had a hard time of it. My children were extremely pettish and peevish, and what with nocturnal calls, I had not a night's rest for five years. If anything ailed them, they were sure to cry the night long; but if they were well, they woke up long before the crowing of the cock, climbing over me at the very moment when I had composed my head for a short morning nap. But paternal philosophy can well be reconciled to the sweet music of "crying babes," some thousands of which have been imported into New York during the present year. But the number of people taken sick in the day-time, who send for the doctor at night, produced a compound fracture of my time, which seldom gave me a comatose state. It is the sweetest of all consolations to lay a weary head upon the pillow, with the thought that rest awaits you until the dawning light. Whatever carking cares have vexed you, that is a long season of immunity which stretches through the dark hours of the night. Then do the strained muscles lapse into the most easy attitudes in the yielding couch, and the taxed intellect is still, and you bolt the door upon ingratitude and strife.

But to lie down without security from disturbance is enough to frighten away sleep. Such is the lot of a country doctor. I could relate innumerable instances of the utter disregard with which he is routed from his bed, without occasion, at all hours. Here is one in point:

I arrived late one winter evening at my own door, after a hard day's toil.

With what a feeling of relaxation did I divest my feet of heavy boots, set them smoking at the fire, and then regale them in easy slippers! Then, wrapping about me a soft padded gown, with what luxury did I fall back in my arm-chair, peruse the daily paper, and sip a cup of tea! "Now," said I, "the labors of the day are over. A storm is brewing out of doors. I hope that no body will come here to-night. If they do, I won't go. Let them go after Bogardus. I won't immolate myself for any body. It is unreasonable." With that I pulled down my ledger and made a note of the day's visits, one half of which were to poor houses, negro huts, and Irish shanties. As to this class, they loved me like a brother, and their confidence in me was unbounded. They sent for me if their bones ached, or if their corns hurt them, and I went with all speed, though I sometimes had occasion to scold them. Before retiring for the night, I opened the outer door, as was my custom, to see the state of the weather. It was a tremendous night. The moon shone palely, but the wind blew a hurricane. It rained, it hailed, it snowed, it blowed. I thought again of the poor mariners on the coast, and with a silent prayer for them, and all houseless, unprotected ones, I closed the door, and went to bed. I had just recovered from the shivering sensation of cold sheets, and become conscious of grateful warmth, while that delightful drowsiness which borders upon sound sleep stole over me, when there came a knocking, impatiently repeated, enough to wake the dead. "Bless me!" I groaned out, crawling out of bed, and lifting the sash, "what do you want?"

"Doctor, want you to come right straight away off to Banks's. His child's dead."

"Then why do you come?"

"He 's p'isoned. They gin him laud'num for paregoricky."

"How much have they given him?"

"Dono. A great deal. Think he won't get over it."

"When did they give it to him?"

"This arternoon."

"Why did n't you come sooner? How do you think I am to go two miles on such a night? Have you brought a wagon?"

"No."

"Then I won't go. Tell them to ———;" and having prescribed hastily out of the window, I closed the sash, and went back to bed. But the howling wind and rattling sleet against the panes had not that soothing effect which they have to one who lies snug and warm and irresponsible in his couch. "What," said I, "if that child should die through my neglect! Will it absolve me from criminality because the parents are poor? I will go: I must." With that I leaped out again, kindled a match, and went down into my office. Not choosing to wake my man Flummery, or to disturb my old horse, who was craunching his oats, and housed for the night, I took my stick, and set out to walk. The snow water went through my shoes like a sieve; my neck and bosom were instantly covered with sleet. Nevertheless, I had some humorous thoughts while breasting the storm, and composed a Latin distich by the way. I had just got the last foot of the pentameter correct, when my own foot struck against something which looked like a black log. On scrutiny, by the light of the moon, I found it to be my old patient, Timmy Timmons, apparently sound asleep, with his beloved rum-jug by his side. In vain I shook him, to make him aware of his situation, and see if the spirit had left his body. I shook the rum-jug, but there was no spirit there, not a drop. "Timmy," said I, "wake up." No answer. I then kicked him, but he bore it as if he had been used to kicks. "He is dead," said I, and passed on to the next house. There, while opening the gate, I was fiercely attacked by a stout bull-dog; and while keeping him off, and fighting my way up to the house, the master came out in his shirt-tail with a loaded gun. "Do n't you know me?" said I, as he examined the priming; "it is the doctor."

"Souls alive!" responded he; "I thought it was a thief! I'm glad you spoke when you did. In a minute more I should have popped you over, Doc'. Sorry to do that. My son John 's got the fever-aig. Here, Bull, Bull, Bull, Bull!—g' home, sir!"

"Timmy Timmons," said I, "is lying out in the lane, drunk or dead, I do n't know which; dead drunk at any rate. He must be looked after."

"Wait till I put on my breeches. What a wonnerful night! Won't you come in and get warm?"

"No; get on your breeches and make haste."

"Guy! when I first heered you, I thought it was Lawrence comin' to break house. He 's a desput fellow. So I gets up and looks out of the window, and then I went into the corner to find my gun, and if I did n't——"

"Come, come; do you want——"

"To get the rheumatiz? No, I do n't. Hold on, Doctor; be down in one minute."

We returned to the congealed Timmons. My coadjutor took up the jug, shook it, and said, "Not a drop." He then smelt it.

"It is rum," said I, "the cause of all this misery."

"No, Doctor, not *all* rum; there 's been a little *molasses* into the jug, by the smell of it."

"Lift him up," said I. He did so, and carried his burden home, where I brought Timmy to life.

I now trudged on upon my original errand, hoping to save another life more valuable than that of Timmons. Arrived at the house, I perceived it shut up as if hermetically sealed. Not a light was to be seen. I knocked furiously, and at last a night-cap appeared from the chamber window, and a woman's voice squeaked out, "Who 's there?"

"The doctor, to be sure," said I; "you sent for him. What the dogs is the matter?"

"Oh, it 's *no* matter, Doctor. Ephraim's better. We got a little *skeered*, kind of. Gin him laud'num, and he slept kind o' sound, but he 's woke up now."

"How much laudanum did he swallow?"

"Only two drops," said she. "'T as n't hurt him none. Wunnerful bad storm to-night!"

I buttoned my coat up to my throat, turned upon my heel, and tried to whistle.

"Doctor, Doctor."

"What do you want?"

"You won't charge nothin' for this visit, will you?"

Now, as I traveled back on foot, the moon became obscured, the driving sleet blinded the eyes, I heard the Atlantic breakers booming and beating upon the coast; and with head down, like a bulrush, I arrived at my own door, wet and disconsolate, saying to myself, "THAT LITTLE PLANT CALLED PATIENCE DOES NOT GROW IN EVERY GARDEN!"—*New Hampshire Journ. of Medicine.*

